# Unifying perspectives on the activity and genotypic targeting of pharmacological chaperones

**DOI:** 10.1016/j.jbc.2025.110375

**Published:** 2025-06-18

**Authors:** Austin Tedman, Muskan Goel, Sohan Shah, Jonathan P. Schlebach

**Affiliations:** The James Tarpo Jr and Margaret Tarpo Department of Chemistry, Purdue University, West Lafayette, Indiana, USA

**Keywords:** pharmacochaperones, correctors, protein misfolding, proteostasis, CFTR, rhodopsin, membrane protein folding

## Abstract

Several diseases of protein misfolding can now be treated with an emerging class of therapeutics known as pharmacological chaperones, pharmacochaperones, or correctors. These small molecules exploit the universal thermodynamic coupling between ligand binding and protein folding to suppress conformational defects that disrupt protein homeostasis. While the mechanistic basis of their activity is quite simple in theory, their nuanced proteostatic effects can vary depending on the intrinsic properties of their target proteins and the cellular context. Deviations in activity are especially pronounced across panels of pathogenic variants of the target protein. In this perspective, we explore the factors that shape the potency of pharmacochaperones and the intrinsic sensitivity of different target proteins in relation to various theoretical considerations and experimental observations. We discuss how emerging technologies have provided general insights into the molecular basis of the variant-specific effects of certain pharmacochaperones. We also highlight ongoing efforts to identify existing drugs that stabilize misfolded variants and to repurpose them as pharmacochaperones. Finally, we discuss how the chaperone activity of current drugs could potentially contribute to complex pharmacology and deviations in therapeutic efficacy across patient cohorts. Together, these principles provide a coherent framework that may help guide the discovery and precision targeting of next-generation pharmacochaperones for both current and new targets involved in proteostasis diseases. An audio recording of this work is included in the supplement and can be freely streamed here.

## Main Text

Be they cofactors, allosteric modulators, inhibitors, or substrates, the coordination of small molecules has a strong influence on the conformational ensembles of a substantial fraction of the proteome (1, 2). Such ligands typically modulate the activities of their target protein by binding to and stabilizing specific active or inactive conformations. But regardless of whether these molecules enhance or suppress activity, they all stabilize the ensemble of natively folded conformations relative to the vast array of competing misfolded structures, which typically lack ordered binding pockets. As a result, ligand binding can often suppress misfolding and degradation by cellular quality control (QC) machinery (3). These considerations provide a framework for understanding how small-molecule therapeutics known as pharmacological chaperones, pharmacochaperones, or correctors can rectify pathogenic imbalances in protein homeostasis. Although pharmacochaperones are currently used for the treatment of several genetic diseases (4), they typically exhibit variations in therapeutic efficacy among patients bearing mutations that cause distinct molecular defects in the target protein (5, 6). An understanding of the mechanistic basis of these variant-specific effects may help to develop broadly efficacious pharmacochaperones and to target them to correctable patient genotypes (7). In the following, we explore the mechanistic effects of current and forthcoming small-molecule pharmacochaperones as well as recent insights into the nature of their variant-specific effects. We also explore how the general linkage between binding and folding equilibria may potentially factor into the effects of certain traditional drugs. Based on these considerations, we outline potential opportunities to leverage recent advances for the development of new pharmacochaperones, to repurpose existing drugs for proteostasis, and to target these compounds to the subsets of misfolded protein variants that are amenable to therapeutic rescue.

## Clinical emergence of pharmacological chaperones

Since pharmacological chaperones were initially conceptualized over 20 years ago (8, 9, 10) a variety of promising compounds have entered clinical trials, and several are now approved for the clinical treatment of various protein misfolding diseases. Within the past decade, at least eight pharmacochaperones have received FDA approval for the treatment of four distinct diseases of aberrant proteostasis (Table 1). Although each of these drugs targets proteins that undergo pathogenic misfolding, the underlying pathophysiology of these diseases has little in common. The collective success of these drugs speaks to the generality of this strategy. Indeed, each of these druggable target proteins relies on distinct biosynthetic assembly factors and possesses distinct structural features that shape their sensitivity to stabilizing small molecules. For instance, the sickle cell anemia drug GBT440 (11) suppresses hemoglobin aggregation by selectively stabilizing the oxygenated heterotetramer (12), the assembly of which relies on a dedicated molecular chaperone (AHSP), the coordination of heme cofactors, and the maintenance of a stoichiometrically balanced pool of α and β subunits. In contrast, the suppression of α-galactosidase A (α-GAL A) misfolding by Migalastat, an approved treatment for Fabry disease, must play out in a distinct context given that this protein is secreted through a translocon into the oxidizing environment of the ER lumen where it is engaged by chaperones that coordinate its folding and modulate its trafficking to the acidic lysosomal lumen. These processes are fairly simple relative to those that coordinate the assembly of the cystic fibrosis transmembrane conductance regulator ion channel (CFTR), a target for leading cystic fibrosis (CF) drugs that is cotranslationally folded into ER membranes while interacting with dozens of QC proteins (13, 14). How, when, and where does the binding of stabilizing small molecules impact these processes, and how does this stabilization alter the overall balance between folding and degradation? Can these considerations be generalized and/or used to predict optimal pharmacochaperone targets? In the following, we will explore these questions in the context of emerging insights into the mechanistic effects of pharmacochaperones.

**Table 1:** List of pharmacochaperones in the clinic or in clinical trials

Compound	Status	Target protein	Disease
VX-809	Approved	CFTR	Cystic Fibrosis
VX-661	Approved	CFTR	Cystic Fibrosis
VX-445	Approved	CFTR	Cystic Fibrosis
VX-121	Approved	CFTR	Cystic Fibrosis
SION-2222	Phase II	CFTR	Cystic Fibrosis
SION-2851	Phase I	CFTR	Cystic Fibrosis
SION-109	Phase I	CFTR	Cystic Fibrosis
SION-638	Phase I	CFTR	Cystic Fibrosis
SION-719	Phase I	CFTR	Cystic Fibrosis
GBT440	Approved	Hemoglobin	Sickle Cell Anemia
Tafamidis	Approved	Transthyretin	TTR Amyloidosis
Acoramidis	Approved	Transthyretin	TTR Amyloidosis
Migalastat	Approved	α-galactosidase A	Fabry Disease

## Energetic constraints on pharmacochaperone activity

Regardless of where pharmacochaperones engage their targets in the cell or at what point(s) in the protein life cycle this occurs, there is one thing these molecules all have in common-they all specifically bind and stabilize the native fold of the target protein. From first thermodynamic principles, how ligand binding enhances the protein stability can be understood with the following simplified, generalizable relationship previously derived by Park & Marqusee (15).

(1) $${\Delta G}_{app}=\Delta G{}^{\circ}+RTln\left(1+\frac{\left[L\right]}{\left[K_{d}\right]}\right)$$

where *ΔG*_*app*_ represents the apparent free energy of folding in the presence of ligand, *ΔG°* represents the free energy of folding in the absence of ligand, R is the gas constant, T is the temperature, *K*_d_ is the equilibrium dissociation constant of the ligand, and [L] is the free concentration of ligand. The degree to which a ligand increases protein stability is captured by the second term on the right side of the equation, which shows stabilization can be maximized by either increasing the binding affinity (lowering *K*_d_) or increasing the concentration of bioavailable ligand. The beauty of this equation lies in its simplicity and generality-thermodynamic coupling between binding and folding should hold for any class of ligand and any sort of structure, so long as the relevant misfolded conformations do not also bind the ligand. Indeed, the generality of this framework has been borne out by various experimental observations. For instance, a comparison of the proteostatic effects of a series of ligands for the gonadotropin-releasing hormone receptor (GnRHR) revealed that chaperoning was most closely associated with binding affinity across a panel of agonists and antagonists (16). This simplistic reliance on binding energy suggests that pharmacochaperone development may be particularly amenable to rational structure-based drug design (17, 18). With any sort of liganded structure, it may be possible to build high-affinity analogs that endow the target with enhanced affinity. Nevertheless, the overall functional impact of the resulting ligands will ultimately hinge on the balance between their effects on both the expression and the activity of the target. Such tradeoffs are hard to generalize and may require further consideration of the specific aspects of the target, including its intrinsic stability, its folding/assembly kinetics, and/or its precise role in the molecular basis of disease, as we will discuss below.

**Figure 1:**
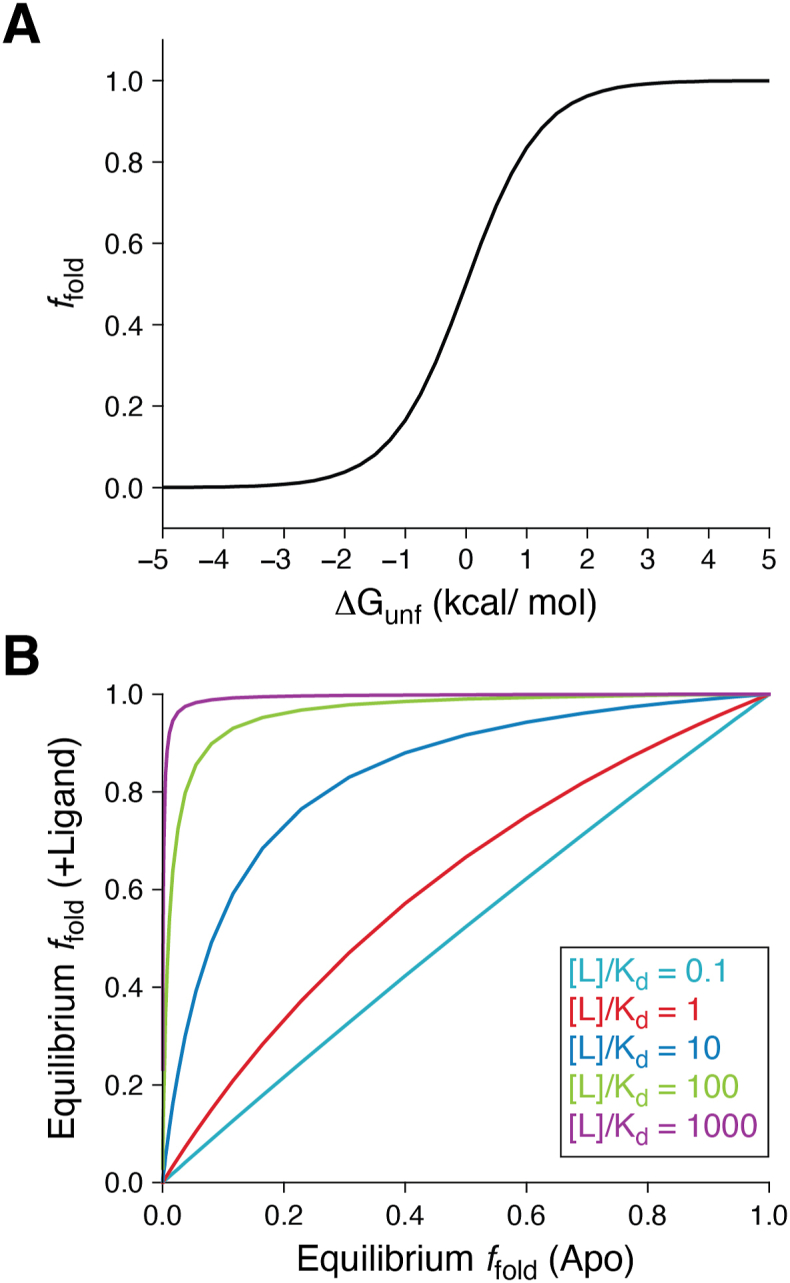
**thermodynamic Coupling Between Folding and Binding.***A*, the equilibrium fraction of folded protein is plotted against the free energy of unfolding for a two-state folding equilibrium. *B*, the equilibrium fraction of folded protein in the presence of a ligand is plotted against the equilibrium fraction of folded protein in the absence of a ligand under various conditions in which the ratio of the ligand concentration to the equilibrium dissociation constant is varied. Figure *panelB* was originally published in Reference (25).

There are, of course, caveats and practical limitations to these energetic considerations. While drugs can be modified to enhance their bioavailability and/or pharmacokinetics, no amount of optimization will result in the accumulation of mM quantities of free drug under physiological conditions. Likewise, with few exceptions (19), modifications to the structures of lead compounds typically cannot produce sub-nanomolar equilibrium dissociation constants. These two factors generally constrain the degree to which a single pharmacochaperone can shift the folding equilibrium of its target protein. In light of this consideration, it is perhaps no small coincidence that leading clinical CF drugs such as Trikafta (20, 21) contain combinations of stabilizing pharmacochaperones that bind distinct pockets in order to achieve greater rescue of misfolded CFTR variants (22). Clearly, in this case, two pockets are better than one. Although some exceptions exist (19), it may be generally easier to discover 2 different nM binders that target distinct pockets rather than a single picomolar binder. Assuming the binding of one compound does not allosterically impact the binding of another, combining 2 nM binders should essentially double the binding energy term in Equation 1. Moreover, because of the nonlinear dependence of the fraction of folded protein on the free energy of folding (Fig. 1*A*), combinations of non-redundant pharmacochaperones may result in the synergistic enhancements in expression as is the case for combinations of non-redundant CFTR correctors (*i.e.* VX-661 + VX-445) (23, 24).

How much binding energy is enough? The answer to this question must depend on both the pathophysiology of the specific disease and the biophysical properties of the target. With regard to the latter, Equation 1 can be used to cast the effect of a pharmacochaperone on the fraction of folded protein in terms of the [L] to *K*_*d*_ ratio as well as the equilibrium fraction of folded apo protein as is shown in Figure 1*B* (25). Note that, for any value of [L]/*K*_*d*_, the maximal shift in folding will occur when the folded and unfolded forms of the apoprotein are equally favorable (*f*_fold_ = 0.5, *ΔG°* = 0). Thus, metastable proteins will always be the most sensitive to pharmacochaperones, regardless of the abundance of the pharmacochaperone or the strength of its interaction with the target. Furthermore, for metastable proteins (or protein variants) within this regime, even small amounts of binding energy (*i.e.* the second term in Equation 1) can translate to large shifts in the fraction of folded protein. This is a *universal principle* that emerges from the sigmoidal dependence of the fraction of folded protein on the free energy of folding-the curve is at its steepest when *ΔG°* = 0 (Fig. 1*A*). By comparison, greater binding energies are required to appreciably shift the folding equilibria of highly unstable proteins (or protein variants). Likewise, it is equally difficult to move the needle for highly stable proteins, though there should be little need to target such proteins in this way.

**Figure 2:**
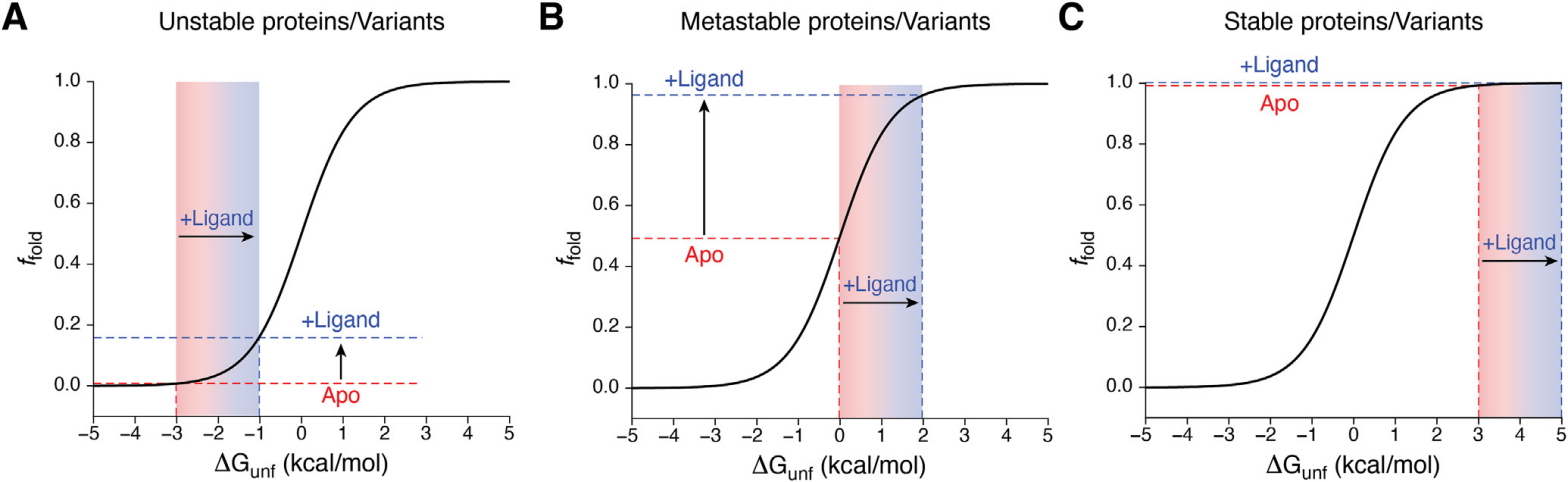
**Impacts of binding energy on the folding of stable and unstable proteins.** Plots of the equilibrium fraction of folded protein (*f*_fold_) against the free energy of unfolding (ΔG_unf_) depict how an arbitrary amount of binding free energy and the resulting shift in the folding equilibrium (*i.e.* ΔΔG_unf_ = +2 kcal/mol) differentially affects the *f*_fold_ of (*A*) unstable (*e.g.* ΔG_unf_ = −3 kcal/mol), (*B*) metastable (*e.g.* ΔG_unf_ = 0 kcal/mol), and (*C*) stable (*e.g.* ΔG_unf_ = +3 kcal/mol) proteins and/or protein variants.

Beyond the properties of the ligand, these considerations suggest certain marginally stable target proteins/protein variants may lie within a “goldilocks” regime that renders their folding highly sensitive to ligand binding (Fig. 2). We note that, while it is true that the native conformations of most proteins are thermodynamically preferred by 5 to 10 kcal/mol, at least 10% of cellular proteomes are intrinsically unstable (26, 27), and there are certainly examples of metastable targets for proteostasis diseases such as peripheral myelin protein 22 (PMP22, *ΔG°* = 0 kcal/mol) (28, 29). There are also many examples of misfolding diseases that involve highly stable target proteins (*e.g.* rhodopsin) that are instead rendered unstable by a spectrum of mutations that compromise folding energetics as is discussed further below. The importance of the intrinsic stability of the target is illustrated by our recent efforts to profile the response of various targets to pharmacochaperones. In a comparison of targets under identical cellular conditions, we previously found that a nanomolar binder generates a ∼30% increase in the expression of rhodopsin (30), a notoriously stable G protein-coupled receptor (GPCR), while nanomolar binders for the misfolding prone CFTR channel can achieve up to a 260% increase in expression (24). Such considerations should factor heavily into the choice of screening targets (or target variants). It may be difficult to find compounds that fix the most unstable (or stable) disease variants. The resulting lack of sensitivity of such screens may therefore miss promising scaffolds that could be developed into high-affinity pharmacochaperones. We also note that, while these thermodynamic perspectives are useful, they may also be insufficient to capture the relevant properties of certain targets and/or the effects of small molecules. For instance, while tight binding is necessary for stabilization, thermodynamic stabilization may not be sufficient to stave off kinetically controlled misfolding and degradation processes. Perspectives on folding energetics must also be weighed against other aspects of pathophysiology, such as the potential perturbation of regulatory modifications and/or the degree of functional rescue that may be required to achieve efficacy. Nevertheless, the simplified perspectives outlined below provide a useful framework to help guide the discovery process.

### Target-specific constraints on pharmacochaperone activity

While there are many known proteostasis diseases associated with unstable proteins (31, 32), there are certain intrinsic protein properties that render some targets more druggable than others. Based on the considerations above, the stability of the target protein and/or target protein variants should factor heavily into drugability. Although accurate prediction of protein stability from sequence and/or structure remains challenging, recent advances in generative modeling have increased the accuracy of such predictions for small, single-domain proteins (33, 34). In some select cases, the stability of the target may be known or readily measurable (35). However, for targets such as integral membrane proteins, thermodynamic parameters describing the relevant folding/misfolding transitions may be difficult to fully conceptualize, much less experimentally measure (36, 37). Nevertheless, beyond the free energy of folding, there are other biochemical properties that may indicate whether a target protein may be sensitive to pharmacochaperones. For instance, many metastable proteins exhibit enhanced expression at lower cellular growth temperatures and recent deep mutational scanning investigations have revealed strong correlations between temperature-sensitivity and the corresponding sensitivity to pharmacochaperones (38, 39). Additionally, many potential targets of interest are already known to coordinate natural cofactors that stabilize their native structures (40). In such cases, nature may already rely on small molecules to modulate stability and/or expression. This sort of target-specific information may suggest tractable pockets and/or molecular scaffolds that can be derivatized to develop high-affinity ligands that act as pharmacochaperones. While pharmacochaperones that target active sites run the risk of inhibiting the target by out-competing the endogenous ligands, substrates, and/or cofactors, there are several examples of inhibitors that also act as effective pharmacochaperones (10, 39, 41, 42, 43). Counterintuitively, these inhibitors do, in fact, restore both the expression *and activity* of certain misfolded variants at sub-inhibitory concentrations (10, 39, 41, 42, 43). Such an outcome suggests the enhanced expression that is achieved in the presence of the pharmacochaperone offsets the coincident inhibition of the rescued enzyme-a partially inhibited enzyme is more active than a misfolded and/or degraded one.

**Figure 3:**
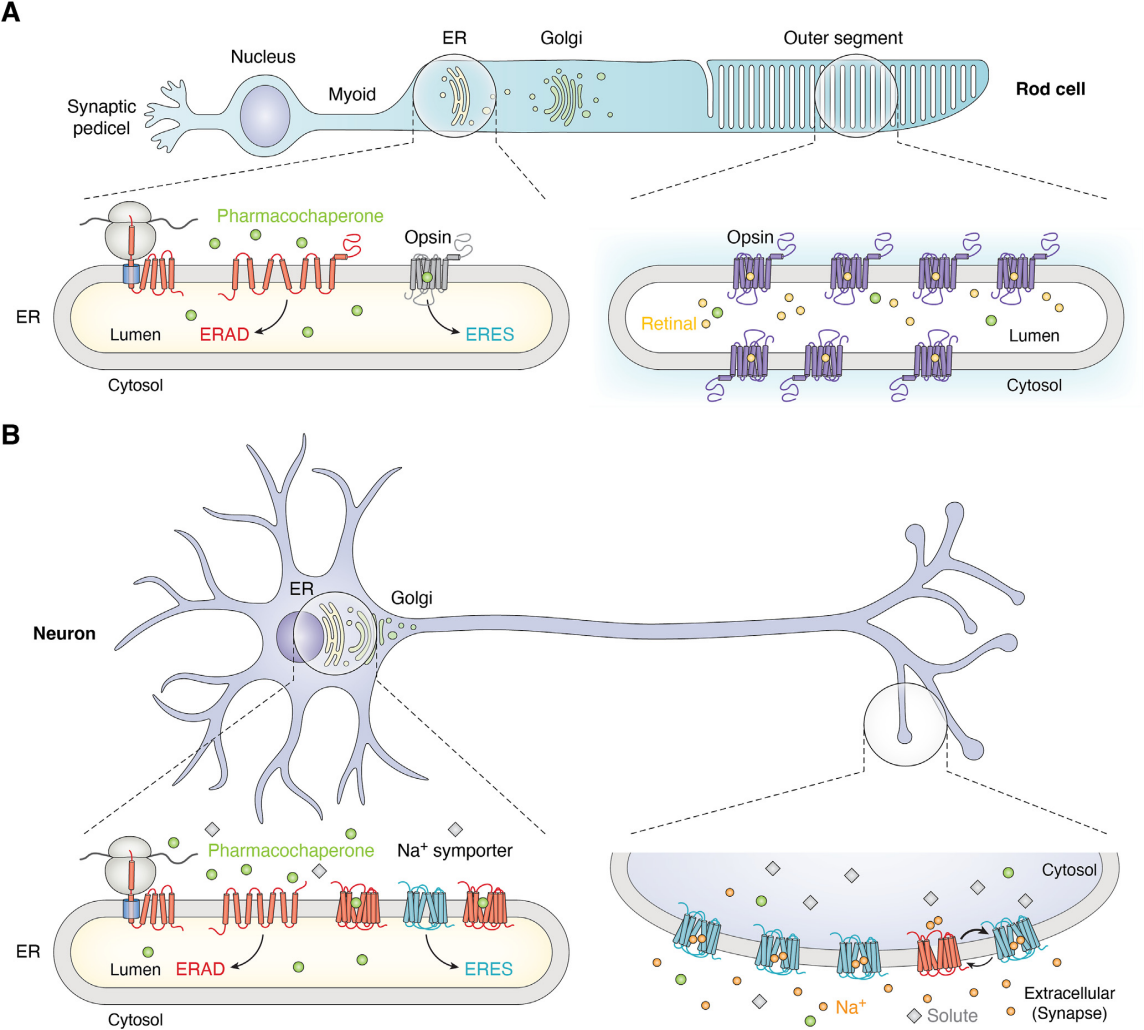
**Physiological context of pharmacochaperone activity.***A*, a cartoon depicts the organization of a rod cell within the retina. Pharmacochaperones may promote opsin folding in a manner that diverts the nascent receptor from the ER-associated degradation pathway (ERAD) to the ER exit sites (ERES). However, the pharmacochaperone is unlikely to outcompete the retinal cofactor in the context of the rod outer segment, where the protein functions. *B*, A cartoon depicts the organization of a neuron. Pharmacochaperones may promote neurotransmitter sodium symporter folding by selectively binding to its inward-facing conformation (*red*) in a manner that diverts the nascent transporter from ERAD to ERES. However, the high concentrations of synaptic sodium are likely to stabilize the outward-facing conformation of the receptor in the pre-synaptic cleft, which may prevent pharmacochaperones from inhibiting the mature protein.

Competitive interactions may be less relevant for proteins that are trafficked between organelles. For instance, the rhodopsin GPCR, a visual receptor expressed in the rod cells of the retina, must bind to a specific isomer of its retinal cofactor to carry out its biological function. Retinal binds extremely tightly to rhodopsin’s orthosteric pocket (*K*_d_ = 25 pM) (44), which endows the complex with immense stability. However, retinal is only delivered to receptors that reside within a specialized cellular compartment known as the rod outer segment (Fig. 3*A*). As this cofactor is not abundant within the secretory pathway, the nascent opsin apoprotein is vulnerable to pathogenic misfolding within the ER. Nanomolar binders that hit the orthosteric pocket can suppress pathogenic rhodopsin misfolding (43, 45, 46, 47, 48). And while these molecules compete with retinal binding *in vitro*, they are unlikely to compete with the tight binding of the native substrate in the context of the outer segment, as evidenced by their efficacy in mouse models (Fig. 3*A*) (43, 45, 47). Nevertheless, such compounds have yet to gain clinical approval. We note that recent attempts to profile comprehensive libraries of retinopathy variants suggest that about half compromise rhodopsin folding and that many of those variants may fail to respond to certain pharmacological chaperones (25, 43, 47). For these reasons, we suspect the prospects for clinical approval of a rhodopsin pharmacochaperone may require clinical trials that are targeted to specific patient genotypes based on an *in vitro* pharmacology data strategy that proved critical for the approval of CFTR modulators (49, 50, 51, 52).

Similar considerations apply to the SLC6 family of neurotransmitter sodium symporters such as the *SLC6A4* serotonin and *SLC6A3* dopamine transporters (53, 54). These proteins cycle between an outward-facing conformation that binds two sodium ions and one solute molecule from the extracellular synaptic cleft, and an inward-facing conformation that releases this cargo into the cell (55). While the 100+ mM sodium in the extracellular fluid/synapse should lock these transporters in their outward-facing conformations, the lack of a comparable gradient across organelle membranes may render the nascent transporters vulnerable to misfolding in the ER (Fig. 3*B*) (56). Most of the stimulants, antidepressants, and other drugs that inhibit these transporters selectively bind the outward-facing conformation that predominates at the plasma membrane (57). However, there are a smaller number of so-called “atypical” inhibitors that instead stabilize their inward-facing conformations (58). This conformational bias renders these molecules more effective at binding the unconstrained nascent transporter in the ER than to the outward-stabilized transporter at the plasma membrane. Importantly, these atypical inhibitors act as pharmacochaperones that can rescue both expression *and function* of unstable transporter variants (41, 42, 59, 60). We note that, based on the fact that such compounds can rescue multiple SLC6 transporters, atypical inhibitors could potentially offer therapeutic utility for the rescue of pathogenic *SLC6A8* creatine transporter variants, the misfolding of which causes an X-linked neurological disorder known as cerebral creatine deficiency syndrome (61, 62, 63, 64). Together these considerations suggest membrane proteins and other secreted proteins that are born in the ER yet evolve to function in other environments may be particularly amenable to rescue with a variety of tight binders including inhibitors.

In addition to functional pockets, many targets also contain other orphan and/or cryptic pockets that do not appear to recognize physiological ligands, yet are nonetheless potentially druggable. After all, just like functional pockets, these structural niches are generally only present when the polypeptide is folded into a relatively small number of well-folded conformations- binding to these pockets should therefore drive folding *via* mass action. In cases where these pockets are defined enough to coordinate a ligand, they also boast the advantage that their saturation is less likely to interfere with functional interactions within active sites. Perhaps the most obvious real-world examples of pharmacochaperones that bind to otherwise inconsequential pockets are the modern CFTR correctors (65), the scaffolds of which were discovered by high-throughput screening. How these molecules bind and stabilize the CFTR protein remained unclear even after they were clinically approved. However, a series of recent cryo-EM structures have revealed that these molecules bind to two previously unknown “pockets” that lie along the surface of their membrane-spanning domains (22, 66, 67, 68, 69). Although these pockets are not particularly deep, these correctors do achieve low nM binding affinities. What’s more, combinations of these compounds can “allosterically” correct folding defects across subdomains because of their additive binding energies (23, 24). The success of this strategy speaks to the advantage of targeting (or happening upon) multiple orphan pockets.

**Figure 4:**
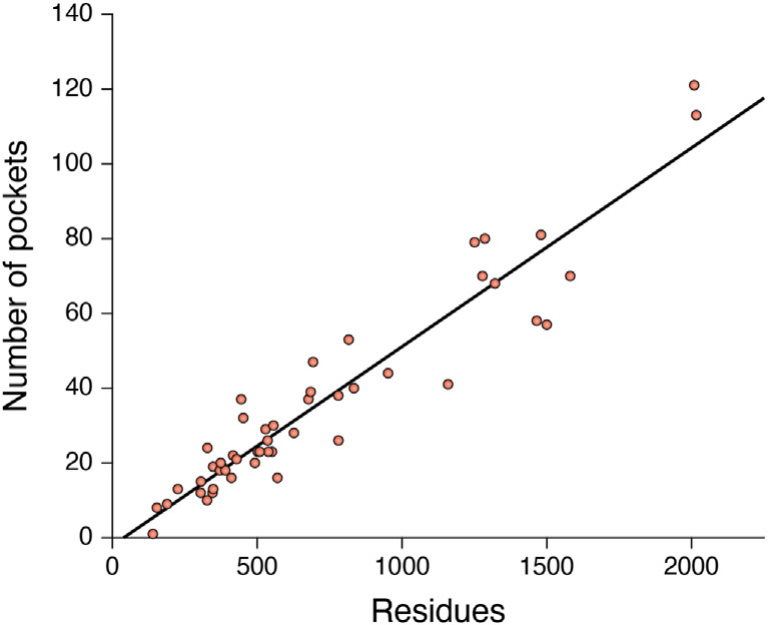
**Druggability of pharmacochaperone targets.** Autosite was used to identify potentially druggable pockets within structural models of 50 potential target proteins associated with diseases of aberrant proteostasis. The number of pockets identified within each structure is plotted against the corresponding number of residues within the target protein. A linear fit of the data is shown for reference (*R*^2^ = 0.90).

Based on these considerations, proteins featuring a variety of pockets within their native structure, be they functional, allosteric, or orphan, make attractive targets for the rational design of pharmacochaperones. From a medicinal chemistry perspective, larger proteins may have more druggable “real estate” relative to more compact proteins. Many such targets were long forsaken due to the biochemical and structural challenges that come with large, unstable proteins/protein complexes. However, in this golden age of protein structure prediction, it may be worth revisiting rational design efforts targeted to large proteins and/or oligomeric assemblies. Indeed, recent indications suggest AlphaFold2 models may be highly useful for virtual screening-based discovery efforts (70). To explore this concept further, we used AutoSite (71) to identify potentially druggable pockets within a collection of 50 AlphaFold2 models for target proteins associated with various diseases of aberrant proteostasis (Table S1). Our results reveal that the number of pockets within the native structures of these targets is proportional to protein length (Fig. 4). These results suggest larger proteins represent more favorable targets. Indeed, there appear to be many structurally diverse pockets within the native CFTR structure that can be successfully targeted for the development of pharmacochaperones, and successful attempts to target these pockets is fueling the development of a diverse panel of therapeutic small molecules (Table 1). These considerations suggest larger target proteins with complex assembly pathways may be the most fruitful targets for pharmacochaperone development. Together, these principles may help refine ongoing pharmacochaperones discovery efforts that rely on high-throughput screening, structure-based drug design, or some combination of the two.

#### Kinetic constraints on pharmacochaperone activity

Although the energetic perspectives outlined above may capture some of the operative concepts that drive pharmacochaperone activity, the protein biosynthesis and degradation pathways these compounds modulate are largely subject to kinetic control. Kinetic constraints on pharmacochaperone activity are perhaps most pronounced during the pioneering round of protein folding that occurs during translation on the ribosome. While some proteins may rapidly fold and unfold thousands of times during their lifetimes, the slow rate of protein translation (∼5 AA/s) renders nascent polypeptides vulnerable to spurious interactions that can form before the completion of synthesis and/or folding. Based on this consideration, we again expect protein length to factor heavily into biological folding efficiency- the biosynthesis and folding of larger proteins may often involve complex assembly coordinates that play out over long time scales. Consider again the case of CFTR. At 1485 residues, translation of a single CFTR protein should require between 5 and 6 minutes. Certain features of its native structure, such as its domain-swapped interhelical contacts (68), cannot form until several minutes into synthesis. A sizable amount of biochemical evidence suggests that certain CFTR subdomains are vectorially coupled during synthesis (72, 73, 74), although we suspect such interfaces are unlikely to be fully native. The importance of this coordinate is evidenced by the abundance of CF mutations within the first two subdomains that enhance cellular misfolding (24, 75). Indeed, while many misfolded variants can be partially rescued by a pharmacological chaperone that binds within the C-terminal domains during the later stages of assembly, rescue by a compound that binds with a similar affinity to the N-terminal domains in the early stages of assembly is significantly more efficient (24). Therefore, in this case, the point in biosynthesis at which the pharmacochaperone binds the nascent chain appears to factor into the efficiency of pharmacological rescue.

The importance of folding/assembly kinetics is also evidenced by interactome measurements of CFTR, which have revealed how misfolding dramatically reshapes the assembly coordinate. For instance, the misfolding of ΔF508 CFTR coincides with the formation of 200+ aberrant protein interactions, many of which are associated with ER quality control (76). Given the mechanism of cotranslational ΔF508 misfolding (77), many of these ER interactions are undoubtably prompted by its failure to undergo proper cotranslational assembly on a relevant time scale. Recent investigations by the Plate lab have revealed that efficient pharmacological rescue of several CF variants coincides with a partial suppression of certain aberrant interactions. Interestingly, these investigations have shown that the suppression of misfolding by VX-445 appears to stem from the re-balancing of translational interactors while rescue by VX-809 coincides with the restoration of downstream autophagocytic interactions (78, 79). We note that knocking down the expression of certain interactors with distinct proteostatic functionalities can restore the expression of misfolded CF variants (76, 79, 80, 81, 82), implying that the proteostasis machinery collectively works to suppress the biogenesis and/or cellular accumulation of defective CF variants (83). But the biochemical basis of this cellular misassembly process is likely to be a complex, stochastic process that is not fully understood. Regardless, the fact that it forms interactions with proteins found within a variety of organelles serves as a reminder that, at any given point of time, immature CFTR proteins can be found throughout the secretory pathway while the mature protein exists at the plasma membrane. Soluble proteins that are secreted or targeted to organellar lumens are also likely to be found within several compartments at steady state, as their localization hinges on the amount of the flux through the pathway and the fidelity of cellular sorting machineries that are sub-stoichiometric with respect to ribosomes. Thus, the aggregate effects of pharmacochaperones must arise from their collective actions at all points in the protein life cycle. Generally speaking, we suspect the impact of pharmacochaperones on the reactions involved in the initial pioneering round of assembly may be more rooted in the kinetic correction while the influence of these compounds on fully synthesized/assembled proteins within other organelles may be more thermodynamic in nature. The net proteostatic effects of the compound may arise from a combination of the two.

While we have focused these discussions on the well-characterized CFTR protein, we note that there are a variety of additional indications suggesting that kinetic constraints factor heavily into the folding efficiency of various other protein targets. For instance, a previous investigation of the relationship between transthyretin (TTR) folding and secretion revealed that both the kinetic and energetic effects of mutations on its folding and oligomerization must be accounted for in order to recapitulate the relative secretion efficiencies of these pathogenic variants (84). Assembly kinetics may also involve endogenous ligand binding events, as certain misfolded rhodopsin variants that can be functionally rescued by stabilizing retinal cofactors in the cell cannot be refolded by these molecules *in vitro* (38, 85). Taken together, the effects of pharmacochaperones on both the kinetics and energetics of protein folding are likely relevant to the proteostatic balance that is achieved in the cell. We suspect the relative importance of these factors is likely to vary based on the intrinsic stability of the protein target (Fig. 2), the features of its biosynthetic assembly coordinate, and the nature of its cellular context.

### Modulation of pathogenic variants by pharmacochaperones

While certain proteins are more sensitive to pharmacochaperones than others, it is important to consider how the properties of these targets may be distorted by various classes of mutations that disrupt proteostasis. Many coding mutations perturb the energetics of binding and/or folding, which can lead to considerable heterogeneity in their sensitivity to pharmacological chaperones (23, 24, 25, 43, 47). For this reason, the molecular effects of certain mutations and their relative abundance in the clinical population (*i.e.,* penetrance) can play an outsized role in the clinical potential of a pharmacological chaperone.

Early indications that clinical mutations associated with various diseases give rise to diverse molecular effects spurred widespread efforts to classify variants according to the biochemical basis of their fitness effects. The characterization of individual variants over the course of several decades eventually revealed the many ways that mutations can break certain proteins (6, 86). There are, of course, many non-coding mutations can reduce the abundance of functional protein, such as those that compromise transcription or splicing. There are also coding mutations that can reduce expression and/or activity without perturbing protein structure or folding, such as those that alter regulatory modifications. Patients bearing such mutations are unlikely to benefit from pharmacochaperones. However, most pathogenic mutations within coding regions seem to compromise protein function by enhancing misfolding, although these same mutations typically also have secondary effects on the structure and function of the folded form (23, 24, 25, 39). Parsing mutations according to such criterion offers a key advantage in clinical trials (87). Small molecules that enhance function are useless against mis-localized and/or degraded variants. Likewise, pharmacochaperones may have limited impact on variants that are folded but non-functional. Variant classification information ultimately proved invaluable for the development of successful clinical trials for CFTR modulators and their eventual approval as CF therapeutics (49, 50, 51, 52). Targeting combinations of stabilizing molecules and activating “potentiator” molecules to certain classes of misfolded CFTR variants has proven wildly successful in the clinic (20, 49, 50, 51, 52).

The CF success story provides important lessons concerning the development and targeting of pharmacochaperones. As a large multidomain protein that is intrinsically prone to misfolding, the wild-type CFTR protein is highly sensitive to pharmacochaperones-a bellwether for the wider population of CFTR variants. However, most people with CF are homozygous or heterozygous for the ΔF508 variant, which is retained/degraded in the ER and exhibits poor function at the plasma membrane (88). All indications are that this variant lies below the “goldilocks” regime—its high degree of destabilization as well as its poor function at the plasma membrane make it a challenging target to rescue with a single compound. For this reason, leading therapeutics consist of cocktails containing multiple correctors in combination with a potentiator (6, 65). While the robust clinical rescue of this variant using three compounds required additional medicinal chemistry and multiple clinical trials, the eventual success of these therapeutics continue to have a massive clinical impact since the rescue of ΔF508 (and various other rare CF variants) provide a clinical benefit for upwards of ∼85% of people with CF (∼85,000 people worldwide). Unfortunately, the remaining ∼15% of people with CF carrying pairs of rare CFTR variants exhibit wide variations in their response to current CFTR modulator therapies. This variability fundamentally arises because of the diverse molecular defects caused by the spectrum of clinical CF mutations (6). Identifying therapeutic avenues for these people will require solutions for both immediate clinical challenges as well as longer-term logistical “precision medicine” issues.

**Figure 5:**
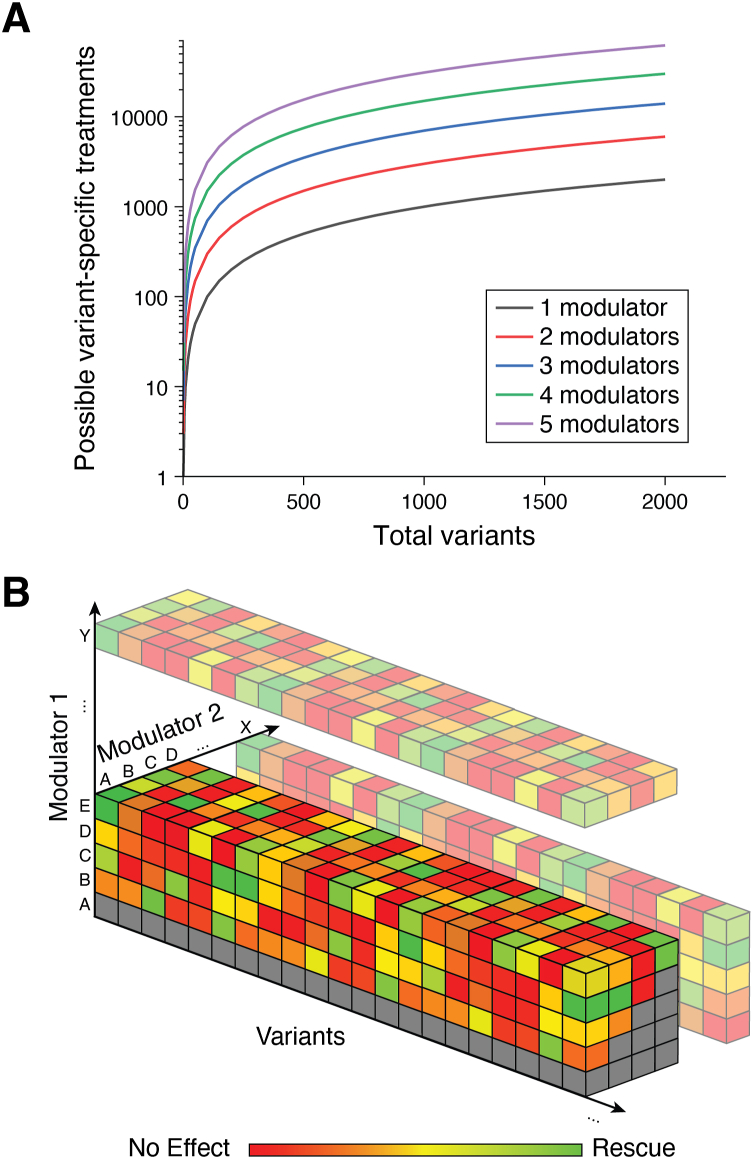
**Expanding Combinatorial Matrix of Potential Targeted CFTR Modulator Therapies.***A,* the number of possible unique variant-specific combinatorial treatments is plotted as a function of total pathogenic variants for treatments consisting of a different number of small-molecule modulators. *B*, a cartoon depicts how the number of possible pairwise combinatorial therapies expands each time a new modulator is approved.

Immediate issues stem from the fact that only some of the 600+ known CF variants can be rescued by current therapeutics (5, 24). An ideal clinical solution for these people would involve a more broadly efficacious therapeutic that is equally effective towards all types of damaged channel proteins. A more realistic approach may involve efforts to match combinations of correctors to specific CF variants based on their pharmacological properties. While leading therapeutic treatments such as Trikafta simply combine the most efficacious versions of the approved CFTR modulators, many people with CF are intolerant of certain compounds. Ideal matching would require comprehensive knowledge of the sensitivity of individual CF variants to all possible combinations of therapeutics (*i.e.* theratype) (6). However, the growing list of known CF variants in conjunction with the increasing number of clinically approved CFTR modulators is fueling a rapid expansion in the potential number of variant-specific CF treatments (Fig. 5)—a long-term precision medicine problem (7). Fortunately, streamlined approaches to efficiently measure the response of individual CF variants to combinations of correctors are providing scalable theratyping solutions. Importantly, these experimental platforms have an opportunity to create real world impact in light of the US Food and Drug Administration policy allowing certain *in vitro* methodologies to be used to indicate CFTR modulator treatments for the clinical treatment of people bearing specific CF variants (87). These emerging methods may help fuel ongoing efforts to “expand the label” of approved modulators to include additional rare CF variants—a critical criterion to ensure coverage of the high costs of these medications (>$300k/year) by US health insurance companies. The importance of this issue is evidenced by the Vertex Pharmaceuticals CF website, where people can enter their CFTR variants in order to determine whether there is sufficient evidence to justify prescription (https://www.vertextreatmentshcp.com/).

**Figure 6:**
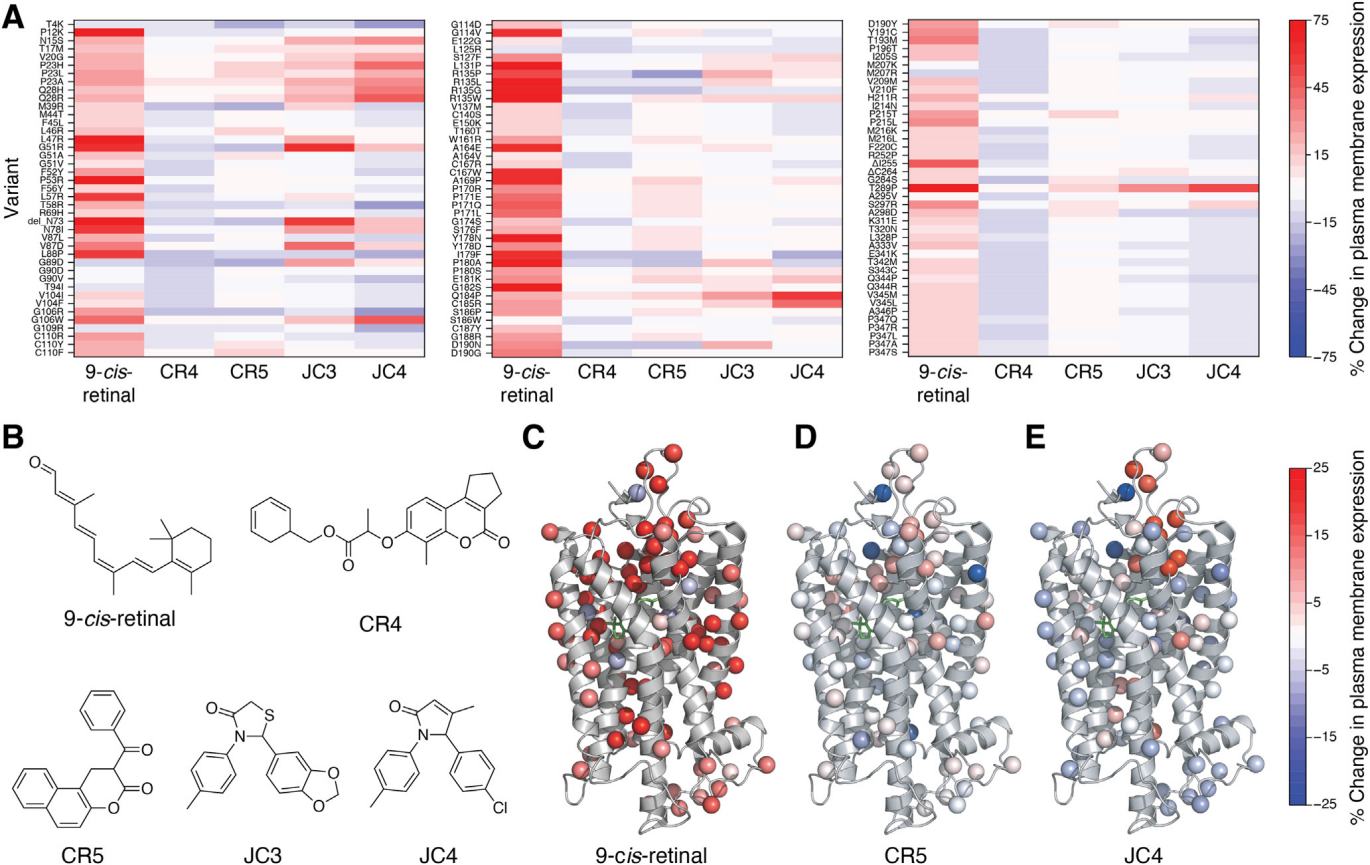
**Variant-specific effects of emerging rhodopsin pharmacochaperones.** The effects of various investigational pharmacochaperones on the plasma membrane expression of 123 rhodopsin variants associated with clinical retinopathies was compared by deep mutational scanning (DMS). *A*, three heatmaps depicts DMS measurements of the change in the plasma membrane expression for 123 pathogenic rhodopsin variants (y-coordinate) in HEK293T cells in the presence of five different investigational corrector molecules (x-coordinate), which were dosed at 10 μM overnight. Changes were measured relative to variant intensity values in the presence of vehicle. *B*, the chemical structures of the five investigational pharmacochaperones are shown for reference. *C,* measured changes in variant expression in the presence of 9-cis-retinal are projected onto the Cα atom of mutated residues within a structural model of rhodopsin (PDB 1U19). *D*, measured changes in variant expression in the presence of CR5 are projected onto the Cα atom of mutated residues within a structural model of rhodopsin (PDB 1U19). *C* and *E*, measured changes in variant expression in the presence of JC4 are projected onto the Cα atom of mutated residues within a structural model of rhodopsin (PDB 1U19). *Red* indicates an increase in variant expression, white indicates no change, and *blue* indicates a decrease in expression. Data and figures adapted from references 25, 43, and 47.

Many of the conceptual advances involved in the successful development of precision CF therapeutics can be directly translated to a host of other genetic diseases associated with large arrays of pathogenic mutations. Alhough there are some exceptions (89), the bulk of the mutations associated with such disorders typically cause a loss of function by enhancing misfolding in one way or another. (24, 25, 29, 39, 61). Based on the considerations outlined herein, the challenge therefore lies in the differentiation of the subset of those that can potentially be rescued by stabilizing small molecules. That is, the misfolded variants that lie within the “goldilocks” regime (Fig. 2). We note that, while many screening efforts have identified compounds that rescue specific variants associated with certain diseases of protein misfolding, in all cases we are aware of, such screening efforts have been designed to search large compound libraries for small molecules that rescue a single genetic variant. While this strategy was quite successful for CF because a large proportion of people with CF carry one particularly common variant (ΔF508), other disorders with comparable molecular pathologies do not have a single dominant variant. As a result, such screening efforts tend to identify small molecules that only seem to efficiently rescue a few variants (64). This key limitation may represent a key reason why so many seemingly promising molecules have been abandoned before or during clinical trials. Future efforts to develop more effective pharmacological chaperones should consider profiling screening hits (or combinations of hits) against wider panels of genetic variants before lead optimization. We note that, while such endeavors would traditionally be prohibitively challenging at scale, refined deep mutational scanning methodologies now provide an efficient means to profile the response of hundreds of variants in parallel (24, 25, 39, 43, 47). Indeed, our recent efforts to compare various hits from pharmacochaperone screens against a comprehensive panel of clinical rhodopsin variants have provided rich information on the variant-specific effects of these promising small molecules that bind within their orthosteric pocket (Fig. 6) (25, 43, 47). Interestingly, the emergent pattern for these compounds reveals that there is not necessarily one universal group of variants that can be rescued by all pharmacochaperones. Moreover, rescued variants do not appear to cluster within a specific region of the three-dimensional structure (Fig. 6, *C*–*E*)—potentially as a consequence of cotranslational effects of these compounds (38, 85). Nevertheless, these sorts of data may provide new opportunities for machine learning approaches to predict variant theratypes-a potentially useful tool to guide targeted clinical trials.

#### Reinterpreting traditional pharmacology and drug repurposing

Generally, most structure-based drug design approaches involve the development of small molecules that manipulate enzymatic cycles or tune the balance between active and inactive conformations within the native ensemble. But regardless of the activity of the target structure, mass action dictates that the binding of all of these molecules should stabilize the native fold relative to a larger competing ensemble of conformations that lack the corresponding binding pockets. These considerations suggest that any class of therapeutic ligand could potentially enhance folding and expression of their target protein. However, in the case of CFTR modulators, chronic dosages of the most common CFTR-activating “potentiator” compound (VX-770) are known to reduce the accumulation of the mature protein over time-the opposite effect of “corrector” molecules that target other pockets within this protein (90). The binding site for this compound is known- it associates with a kinked region of TMD8 within the second membrane spanning domain (91). Though not impossible, it seems implausible that its effects on expression arise from preferential, stabilizing interactions between VX-770 and misfolded structures bearing alternative configurations of TMD8. Alternatively, the VX-770-induced attenuation of CFTR expression could potentially arise from its effects on the cellular sorting of the mature protein. For instance, it may be that constitutively activated channel proteins are more readily funneled into the endosomal lysosomal pathway. Although the mechanism for this potentiator-induced attenuation remains unclear, this previous observation serves to illustrate how the divergent regulatory interactions formed by certain native configurations could potentially lead to distinct proteostatic outcomes despite a similar degree of ligand-mediated stabilization of the native ensemble.

If certain drugs impact the expression of their target proteins, would we typically know? Could the proteostatic effects of small molecule therapeutics factor into their efficacy (or lack thereof) in unknown ways? Might genetic and/or age-related variations in the activity of the proteostasis network across patient cohorts modulate the effects of drug binding on target expression and, ultimately, net efficacy? Across the 3000+ FDA approved small molecules that are prescribed in the clinic, we suspect this must be the case in some instances. Indeed, there are many instances of drugs that exhibit variations in efficacy in the context of aging patients, though this is typically attributed to general changes in metabolism. Based on the principles espoused herein, age-related variations in therapeutic efficacy are perhaps most likely for drugs targeting metastable proteins that fall within the “goldilocks” stability regime (Fig. 2). Recent bioanalytical and computational advances may provide new opportunities to make educated guesses as to which sorts of drug targets are most likely to be sensitive to the proteostatic effects of drug binding. For instance, the application of limited proteolysis in conjunction with mass spectrometry has enabled the identification of unstable proteins at the proteomic scale (2, 92, 93). Moreover, repeating such experiments in the presence of common ligands can reveal numerous proteins that are significantly stabilized by small molecules (1, 94). In the absence of such data, emerging computational modeling approaches are beginning to provide coarse sequence and structure-based estimates for the free energy of folding (26, 27, 33, 34). Overall, these tools and perspectives provide a myriad of new tools to understand hidden aspects of pharmacology.

The utility offered by these perspectives is perhaps best illustrated by ongoing efforts to repurpose and/or modify existing drugs that act as pharmacochaperones for the therapeutic correction of misfolded variants of their target proteins. For instance, the previous discovery of the chaperoning activity of “atypical” SLC6 inhibitors such as bupropion (*i.e.* Wellbutrin) (41, 42, 59, 60), which we discussed above, has inspired new medicinal chemistry efforts to rationalize their structure-activity relationships and to develop next-generation small molecules that selectively stabilize the inward-facing conformations of their targets (17, 18). Such small molecules could potentially be useful for the treatment of Dopamine Transporter Deficiency Syndrome (DTDS), a rare genetic form of infantile parkinsonism associated with the misfolding of *SLC6A3* dopamine transporter variants (95). More recently, the Lehner group built upon previous work showing that the pharmacochaperone activity of the hyponatremia drug Tolvaptan (*i.e.* Samsca) (96) can enhance the expression of nearly all misfolded vasopressin receptor 2 (V2R) variants that cause nephrogenic diabetes insipidus (39). Similarly, the Mu group has found that certain benzodiazepines and imidazopyridines act as pharmacochaperones that can rescue both the expression and activity of various misfolded GABA_A_ receptor variants that are known to cause epilepsy (97). We note that repurposing efforts such as these hold many advantages for drug discovery in the context of rare genetic diseases, where small patient cohorts preclude randomized clinical trials and limitations in the size of the potential drug market often deters drug development within the private sector. In such cases, repurposing medications that have already been approved for the treatment of other disorders may potentially bypass these hurdles and significantly reduce development costs.

### Outlook

For many decades, enzymology and biochemical structure-function paradigms have guided efforts to discover therapeutic small molecules that alter protein activity. However, such molecules may have limited utility for the treatment of proteostasis diseases in which the target protein is misfolded and/or prematurely degraded. A recent pivot towards the development of stabilizing small molecules has provided a new play book for the targeting of unstable proteins that undergo misfolding in the cell. The collective successes and failures of these endeavors now suggest a number of general considerations concerning the relevant features of potential target proteins as well as the pathophysiological factors that can shape pharmacochaperone activity. In conjunction with advances in structural modeling and virtual screening, we suspect this progress will pave the way for numerous drug discovery endeavors for protein misfolding diseases that are currently untreatable. These conceptual advances may also suggest new avenues to explore the proteostatic effects of current drugs and how they may factor into therapeutic efficacy.

## Data availability

All data presented are contained within the manuscript.

## Supporting information

This article contains supporting information.

## Conflict of interest

The authors declare that they have no conflicts of interest with the contents of this article.

## Supporting information

Audio Recording
